# Supplementary material for: Applications of a working framework for the measurement of representative learning design in Australian football
Source: PLoS One. 2020 Nov 30;15(11):e0242336. doi: 10.1371/journal.pone.0242336 (PMC7703947; doi:10.1371/journal.pone.0242336)
Supplement: Attachment — Submitted filename: PONE-D-20-14878_reviewer.pdf [file pone.0242336.s002.pdf]

PLOS ONE

Applications of a working framework for the measurement of representative learning design in Australian football

--Manuscript Draft--

**Manuscript Number:**	PONE-D-20-14878
**Article Type:**	Research Article
**Full Title:**	Applications of a working framework for the measurement of representative learning design in Australian football
**Short Title:**	A working framework for the measurement of representative learning designs
**Corresponding Author:**	Peter R Browne<br>Victoria University<br>Footscray, AUSTRALIA
**Keywords:**	association rules; performance analysis; training design; ecological dynamics
**Abstract:**	Representative learning design proposes that a training task should faithfully represent informational constraints present within a competitive environment. To assess the level of representativeness of a training task, the frequency and interaction of constraints should be measured. This study compared constraint interactions and their frequencies in training (match simulations and small sided games) with competition environments in elite Australian football. The extent to which constraints influenced disposal type and effectiveness across environments was also determined. Constraints relating to pressure and time in possession were assessed, alongside disposal effectiveness, through the use of association rules. These rules were then expanded upon to determine how a disposal influenced the effectiveness of the following disposal. Disposal type differed between training and competition environments, with match simulations yielding greater representativeness compared to small sided games. The subsequent disposal was generally more effective in small sided games compared to the match simulations and competition matches. These findings offer insight into the measurement of representative learning designs through the exploration of constraint interactions. The analytical techniques utilised may assist other practitioners with the design and monitoring of training tasks intended to facilitate skill transfer from preparation to competition.
**Order of Authors:**	Peter R Browne
	Carl T Woods
	Alice J Sweeting
	Sam Robertson
**Additional Information:**	
**Question**	**Response**
**Financial Disclosure**<br><br>Enter a financial disclosure statement that describes the sources of funding for the work included in this submission. Review the submission guidelines for detailed requirements. View published research articles from *PLOS ONE* for specific examples.<br><br>This statement is required for submission and **will appear in the published article** if the submission is accepted. Please make	The author(s) received no specific funding for this work.

Powered by Editorial Manager® and ProduXion Manager® from Aries Systems Corporation

sure it is accurate.<br><br>**Unfunded studies**<br>Enter: *The author(s) received no specific funding for this work.*<br><br>**Funded studies**<br>Enter a statement with the following details:<br>• Initials of the authors who received each award<br>• Grant numbers awarded to each author<br>• The full name of each funder<br>• URL of each funder website<br>• Did the sponsors or funders play any role in the study design, data collection and analysis, decision to publish, or preparation of the manuscript?<br>• **NO** - Include this sentence at the end of your statement: *The funders had no role in study design, data collection and analysis, decision to publish, or preparation of the manuscript.*<br>• **YES** - Specify the role(s) played.<br><br>* typeset	
**Competing Interests**<br><br>Use the instructions below to enter a competing interest statement for this submission. On behalf of all authors, disclose any competing interests that could be perceived to bias this work—acknowledging all financial support and any other relevant financial or non-financial competing interests.<br><br>This statement **will appear in the published article** if the submission is accepted. Please make sure it is accurate. View published research articles from *PLOS ONE* for specific examples.	The authors have declared that no competing interests exist.

**NO authors have competing interests**<br><br>Enter: *The authors have declared that no competing interests exist.*<br><br>**Authors with competing interests**<br><br>Enter competing interest details beginning with this statement:<br><br>*I have read the journal's policy and the authors of this manuscript have the following competing interests: [insert competing interests here]*<br><br>* typeset	
**Ethics Statement**<br><br>Enter an ethics statement for this submission. This statement is required if the study involved:<br><br>• Human participants<br>• Human specimens or tissue<br>• Vertebrate animals or cephalopods<br>• Vertebrate embryos or tissues<br>• Field research<br><br>Write "N/A" if the submission does not require an ethics statement.<br><br>General guidance is provided below. Consult the submission guidelines for detailed instructions. **Make sure that all information entered here is included in the Methods section of the manuscript.**	Ethical approval to undertake the study was granted by the Victoria University Ethics Committee (HRE18 – 022).<br><br>Data was analysed anonymously.

Powered by Editorial Manager® and ProduXion Manager® from Aries Systems Corporation

**Format for specific study types**<br><br>**Human Subject Research (involving human participants and/or tissue)**<br>• Give the name of the institutional review board or ethics committee that approved the study<br>• Include the approval number and/or a statement indicating approval of this research<br>• Indicate the form of consent obtained (written/oral) or the reason that consent was not obtained (e.g. the data were analyzed anonymously)<br><br>**Animal Research (involving vertebrate animals, embryos or tissues)**<br>• Provide the name of the Institutional Animal Care and Use Committee (IACUC) or other relevant ethics board that reviewed the study protocol, and indicate whether they approved this research or granted a formal waiver of ethical approval<br>• Include an approval number if one was obtained<br>• If the study involved *non-human primates*, add *additional details* about animal welfare and steps taken to ameliorate suffering<br>• If anesthesia, euthanasia, or any kind of animal sacrifice is part of the study, include briefly which substances and/or methods were applied<br><br>**Field Research**<br><br>Include the following details if this study involves the collection of plant, animal, or other materials from a natural setting:<br>• Field permit number<br>• Name of the institution or relevant body that granted permission	
**Data Availability**<br><br>Authors are required to make all data underlying the findings described fully available, without restriction, and from the time of publication. PLOS allows rare exceptions to address legal and ethical concerns. See the PLOS Data Policy and FAQ for detailed information.	Yes - all data are fully available without restriction

A Data Availability Statement describing where the data can be found is required at submission. Your answers to this question constitute the Data Availability Statement and **will be published in the article**, if accepted.<br><br>**Important:** Stating 'data available on request from the author' is not sufficient. If your data are only available upon request, select 'No' for the first question and explain your exceptional situation in the text box.<br><br>Do the authors confirm that all data underlying the findings described in their manuscript are fully available without restriction?	
**Describe where the data may be found in full sentences. If you are copying our sample text, replace any instances of XXX with the appropriate details.**<br><br>• If the data are **held or will be held in a public repository**, include URLs, accession numbers or DOIs. If this information will only be available after acceptance, indicate this by ticking the box below. For example: *All XXX files are available from the XXX database (accession number(s) XXX, XXX.*).<br>• If the data are all contained **within the manuscript and/or Supporting Information files**, enter the following: *All relevant data are within the manuscript and its Supporting Information files.*<br>• If neither of these applies but you are able to provide **details of access elsewhere**, with or without limitations, please do so. For example:<br><br>*Data cannot be shared publicly because of [XXX]. Data are available from the XXX Institutional Data Access / Ethics Committee (contact via XXX) for researchers who meet the criteria for access to confidential data.*<br><br>*The data underlying the results presented in the study are available from (include the name of the third party*	All relevant data to reproduce the study are available within the manuscript in the Supporting Information files. We have had to de-identify the data and the drill names.

and contact information or URL).<br>• This text is appropriate if the data are owned by a third party and authors do not have permission to share the data.<br><br>* typeset	
Additional data availability information:	

Manuscript

Click here to download Manuscript Working framework for representative design_Manuscript.docx

Applications of a working framework for the measurement of representative learning design in Australian football

Research Type: Original Investigation

Short Title: A working framework for the measurement of representative learning designs

Peter R Browne[1,2], Carl T Woods[1], Alice J Sweeting[1,2], Sam Robertson[1,2]

[1] Institute for Health and Sport (iHeS), Victoria University, Footscray, Victoria, Australia

[2] Western Bulldogs, Footscray, Victoria

Corresponding author: Peter Browne - Institute for Health and Sport (iHeS), Victoria University, Melbourne, Australia

Tel: +61 436 009 480

Email: peter.browne2@live.vu.edu.au

Total word count: 3263

Abstract word count: 192

Number of tables: 3

Number of figures: 3

Abstract

Representative learning design proposes that a training task should faithfully represent informational constraints present within a competitive environment. To assess the level of representativeness of a training task, the frequency and interaction of constraints should be measured. This study compared constraint interactions and their frequencies in training (match simulations and small sided games) with competition environments in elite Australian football. The extent to which constraints influenced disposal type and effectiveness across environments was also determined. Constraints relating to pressure and time in possession were assessed, alongside disposal effectiveness, through the use of association rules. These rules were then expanded upon to determine how a disposal influenced the effectiveness of the following disposal. Disposal type differed between training and competition environments, with match simulations yielding greater representativeness compared to small sided games. The subsequent disposal was generally more effective in small sided games compared to the match simulations and competition matches. These findings offer insight into the measurement of representative learning designs through the exploration of constraint interactions. The analytical techniques utilised may assist other practitioners with the design and monitoring of training tasks intended to facilitate skill transfer from preparation to competition.

Key words: association rules, performance analysis, training design, ecological dynamics

Introduction

A predominant challenge facing sports practitioners is the design and implementation of training environments that represent competition. This approach to training design has been referred to as representative learning design (RLD) (1). Theoretically, RLD advocates for training to consist of constraints (or information sources) that are experienced within competition to maximise the transfer of skill from training to competition(1, 2). Constraints are categorised into: Individual (e.g., physical attributes and emotions), Task (e.g., rules and ground dimensions) and Environmental (e.g., weather and gravity) classes (3, 4). To assist with the design of representative training tasks, practitioners typically record the constraints of a competitive environment to ensure such constraints are designed into training (5). However, understanding how these constraints interact to influence a performer's actions and behaviours is an ongoing challenge for practitioners, given the non-linearity and dynamicity of sports performance (6).

An important feature of a constraints-led approach to training design is the understanding that constraints do not exist in isolation. Rather, they dynamically interact with one another, often in a continuous manner (4, 7). However, the measurement of the dynamic interaction of constraints has been somewhat neglected within the literature (6). Whilst constraints can be collected from training and competition environments, such approaches often overlook constraint interaction and are unable to capture then analyse the complexity of systems in full (8, 9). Recently, the interaction among constraints was examined via machine learning techniques in Australian football (AF) (6, 10). The application of a rules-based approach enables the complexity of RLD to measured, through the identification of key constraint interactions based on both their frequency and their displayed influence on behaviours. An informed RLD is vital for practitioners as how constraints are enacted in training has been suggested to skill development and learning transfer (1, 11-13).

Within many team sports, including AF, small sided games (SSGs) are used as a frequent training modality due to their perceived representativeness of competition matches and ease of constraint manipulation (14, 15). Specifically, SSGs can be used to simulate sub-phases of competition, whilst to some extent, preserve the complex interactions between an athlete and their environment (16-18). Match simulations are another common training strategy within preparation for performance models in team sports, as they afford practitioners with a practice landscape that can simulate scenarios commonly encountered within competition. Match simulations and SSGs are different types of training modalities and thus, the frequency and interaction of constraints may differ.

The primary aim of this study was to compare constraint interactions and their frequencies, between match simulations, SSGs, and competition matches in AF. Secondly, the study aimed to determine the extent to which they influenced disposal type and effectiveness. Thirdly, this study sought to understand the sequential nature of disposals and whether disposal sequences are dependent upon the preceding disposals. By addressing these aims, this study sought to progress the methodology of measurement for RLD in sporting environments.

Methodology

Data was collected from 2018 official matches and 2019 training sessions from one Australian Football League (AFL) club. All 2018 regular season matches were included (n = 22, disposal instances = 3,478). Specific tasks from training sessions were included, consisting of match simulations (n = 13, disposal instances = 1,298) and SSGs (n = 24, disposal instances = 2,677). Ethical approval was granted by the University Human Research Ethics Committee (application number: HRE18-022).

Match footage was provided by Champion Data (Melbourne, Australia, Pty. Ltd.), whilst training tasks were filmed by club staff, from the same perspective as the match footage

(behind the goals and side view). All footage was then subjected to notational analysis via SportsCode (version 11.2.3, Hudl). Constraints collected included: disposal type, pressure, time in possession and disposal effectiveness (Table 1). These constraint types were based upon similar literature (6, 10, 19). The nature of the options for each constraint sampled limited bias in the rule-based approach, as all constraints had the same number of sub-categories (Table 1).

Table 1. Description of constraints sampled, their sub-category, and definition

Constraint sampled	Sub-category	Definition
Disposal Type	Kick	Disposal of the football with any part of the leg below the knee
	Handball	Disposal of the football by hitting it with the clenched fist of one hand, while holding the football with the other
Pressure	Pressure<br>No Pressure	Opposition player defending the ball carrier from any direction
Time in Possession	> 2 sec<br>< 2 sec	Time with ball in possession from receiving the football to disposing of it
Disposal Effectiveness	Effective<br>Ineffective	An effective kick is of more than 40 m to a 50/50 contest or better for the team in possession, or a kick of less than 40 m that results in retained possession

All analyses were undertaken in the R computing environment (version 3.6.1, Vienna, Austria) and included a three-stage process. All code for the following analyses are available on Github (www.github.com/PeterRBrowne). First, association rules were generated for all disposals for match simulation and SSGs and competitive matches. Association rules are a type of machine learning algorithm which can identify underlying and frequent non-linear patterns in a large dataset (20). The '*Arules*' package was used to apply the Apriori algorithm (21) and to measure the association between multiple constraints on disposal efficiency. Minimum support and confidence levels were set at 0.0002 to allow for all possible rules to be generated. The minimum number of variables was set at four to ensure that each coded constraint was

included. The association rules were arbitrarily assigned an alphabetical identity (ID), being then compared by levels of support and confidence (22).

The frequency a Rule ID was then followed by a subsequent Rule ID was then calculated with the *'tidyr'* and *'dplyr'* packages (23, 24). The difference between training and competition frequencies was then calculated. The observed frequency of a third disposal being effective was calculated. This was visualised using a lattice plot, with colour hues to differentiate the observed frequency of an effective disposal. The level of observed frequency of an effective disposal was calculated as the weighted average of the confidence of a Rule ID and the frequency with which three sequential rules occurred.

Results

The association rules with assigned alphabetical ID are presented in Table 2, and the differences in rule frequency (A) and confidence levels (B) are displayed in Fig 1. The lowest support across all three environments was Rule E (0.012), and the largest was Rule G (0.316), with both occurring in the competition environment (Fig 1A). The support levels for match simulation rules were generally more representative of a competitive match, relative to SSGs, based on the constraints measured. Rule G, a pressured handball performed within 2s, showed the largest difference between competition matches and the SSGs (Fig 1A). Levels of support also varied between environments, with Rule G being the most frequent in matches and match simulations, whilst Rule D was the most frequent in SSGs (Fig 1A). With the exception of Rule C, rules corresponding to 'kicks' yielded lower confidence in competition matches relative to SSGs, but higher confidence relative to match simulations (Fig 1B). For rules relating to 'handballs', the confidence was highest in competition matches relative to the training tasks (Fig 1B).

Table 2. Breakdown of each possible association rule and its associated alphabetical ID

ID	Type	Pressure	Time in Possession (seconds)
**A**	Kick	No Pressure	<2
**B**	Kick	No Pressure	>2
**C**	Kick	Pressure	>2
**D**	Kick	Pressure	<2
**E**	Handball	No Pressure	>2
**F**	Handball	No Pressure	<2
**G**	Handball	Pressure	<2
**H**	Handball	Pressure	>2

**** INSERT FIGURE 1 ABOUT HERE ****

The differences between sequential Rule IDs were calculated between training and competition environments (Table 3). Positive values reflect a greater frequency of occurrence within competition matches, whereas negative values indicate greater frequency of occurrence in the training environment. Match simulations were more similar to competition matches, relative to SSGs in levels of support (Table 3A) and confidence (Table 3B). Disposal sequence differed more between competition matches and SSGs, with eight sequences having a greater than a ±20 % difference between environments (Table 3B). Whilst for similar disposal sequences between training and competition environments, both match simulation and SSGs were similar with twelve and eleven sequences having less than ±1 % difference respectively (Table 3).

Table 3. Difference between frequency of second pass following first pass for competition matches and match simulations (A), and competition matches and SSGs (B). Values are expressed as percentage differences (%).

**A**		Second Pass							
		A	B	C	D	E	F	G	H
First Pass	A	-2	9	3	2	1	-6	-6	-2
	B	4	-18	2	9	-3	-3	11	-2
	C	0	7	-9	5	0	-2	1	-2

	D	-1	5	-11	2	0	-4	12	-4
	E	3	-18	13	0	*NA*	8	0	-5
	F	-2	-7	0	6	0	3	3	-3
	G	-7	1	2	-1	-1	-4	11	-2
	H	-8	-3	-2	0	0	-2	23	*NA*

**B**		Second Pass							
		A	B	C	D	E	F	G	H
First Pass	A	-18	4	-4	-7	3	6	16	1
	B	-4	-24	0	6	0	0	23	-1
	C	-3	19	-22	-26	0	3	28	2
	D	-1	13	-20	-26	0	4	28	1
	E	2	-8	-4	2	*NA*	8	2	-2
	F	-7	-2	-1	0	-1	-2	12	0
	G	-2	4	-2	3	0	-3	2	-3
	H	-3	-3	-3	-19	0	5	26	*NA*

Note: Greater negative values (the deeper the orange hue) indicate greater frequency of the rule sequence in the training environment. Larger positive values (the deeper the blue hue) indicate a greater frequency of the rule sequence in the competition environment. *NA* represents where the two rule IDs did not occur sequentially. Values closer to '0' denote closer similarities between training and competition.

Figure 2 depicts the observed frequency of effectiveness of the third disposal following two sequential disposals across competition matches (A), match simulations (B) and SSGs (C). The variation between competition and training environments are visualised through colour hues, in addition to the observed frequency being overlayed. The third disposal in the sequence was more likely to be effective in SGGs, relative to competition matches and match simulation. Specifically, the observed frequency of the third disposal in the sequence being effective ranged from 54 to 89% for competition matches, 49 to 84% for match simulations, compared to 77 to 88% for SSGs (Fig 3). A majority of competition match third disposal effectiveness were above 70%, with only six disposal sequences less than 70% effectiveness. Comparatively, 28 disposal sequences during match simulations resulted in less than 70% effectiveness (Fig 2,3).

**** INSERT FIGURE 2 ABOUT HERE ****

**** INSERT FIGURE 3 ABOUT HERE ****

Discussion

The primary aim of this study was to compare constraint interactions and their frequencies, between match simulations, SSGs, and competition matches in AF. Secondly, it aimed to determine the extent to which they influenced disposal type and effectiveness. Thirdly, this study sought to understand the sequential nature of disposals and whether disposal sequences are dependent upon the preceding disposals. Accordingly, this study aimed to move forward the methodology for the measurement of RLD to move beyond a single instance and account for continuous nature of match events. For example, by extending the rule-based approach from one disposal (10) and sought to understanding concomitantly disposal sequences, and how disposals are dependent on disposal sequences. Results demonstrated that the frequency and confidence of different disposal types and constraint interactions varied between match and training environments. These differences varied depending on the training task, with match simulations yielding a greater level of representativeness to matches relative to SSGs.

The primary and secondary aims sought to compare constraint interactions and their frequencies, between match simulations, SSGs, and competition matches, as well as determine the extent to which they influenced disposal type and effectiveness. This study demonstrates that an understanding of the differences between support and confidence levels of constraint interactions within training and competition environments is an important consideration for the

design of representative training tasks. For example, match simulations generally showed greater similarity to competition matches, with respect to disposal type. However, competition matches incurred a greater frequency of pressured handballs performed within 2s (Rule G), relative to match simulations. These differences between training and competition environments could exist for a number of practical reasons. Notably, the design features of the SSGs could intentionally favour a specific disposal type (e.g. kick), whilst, in general, the training environment could incur less physical pressure relative to a competitive match, given differences in physical exertion and intensity (19). Practitioners could therefore use this information to improve the representativeness of such training tasks by constraining field dimensions within training to increase player density to lead to an increase in physical pressure imposed from an opponent (25, 26).

Due to the intent of the match simulations compared to SSGs, it is unsurprising to note that match simulations were more representative of competition compared to the SSGs. Thus, it is reasonable to suggest that not all training tasks will yield the same level of representativeness, potentially due to their explicit intent. For example, a practitioner may manipulate certain constraints of a SSG to facilitate greater disposal efficiency, reducing representativeness relative to competition, but still achieving the intended task goal. Conversely, a practitioner may want to challenge disposal efficiency within a SSG, by manipulating temporal and spatial constraints, so the training task is harder (with reference to time and space) than what is afforded within competition. Although it is likely that practitioners' do not plan for every training task to express near perfect representativeness, this methodology provides a platform by which 'target' areas could be identified, informing practitioners as to how frequent non-representative actions are performed within practice. Such information could better guide the macro- and meso- structures of practice, ensuring less representative training tasks are coupled with more representative tasks. Furthermore, the

manipulation of between activity differences align with the principles of the periodisation of skill acquisition (27), but emphasise the importance of being able to measure the influence of constraint interaction within training tasks (10). A training task classification systems may be able to aid practitioners in this process to ensure the appropriate tasks are conducted together based on its characteristics and intent (28). However, more research is required as the ideal balance of representative versus non-representative practice to gain the greatest performance benefit in competition is currently unknown.

The third aim sought to explore concomitantly disposal sequences. Differences between the training and competitive performance environments were found when exploring the observed frequency of a third sequential disposal being classified as 'effective'. Understanding concomitant disposal sequences is a key feature of complexity. This is essential for RLD as it enables understanding of not just the current status, but what concomitantly interactions occur after. For matches and match simulations, the observed frequency of an effective third sequential disposal was lower compared to the SSGs. This practice task yielded the highest range of observed frequency for an effective third disposal, likely due to the task design of the SSGs, which may encourage a more continuous, effective, chain of disposal. This could have been intentionally designed within the SSGs through the systematic manipulation of player numbers (task constraint) to favour the offensive team (for example, a SSG consisting of 6 vs. 4). Nonetheless, this analysis demonstrates how a chain of disposals could partially shape future disposal effectiveness. Subsequently, as a result of understanding the influence of disposals on following disposals allows a practitioner can gain further insight into the representativeness of their training environment, guiding the manipulation of constraints to increase or decrease task complexity.

A limitation of this study was that it grouped all SSGs together, despite it being possible that some SSGs had differing task intentions and subsequent challenge points, thus diluting

their representativeness. This may allow for a more complete insight into the representativeness of individual SSGs. Additionally, a limited number of constraints were used to model RLD, and thus the model presented here is a simplistic view of RLD. The sampling of appropriate constraints is an evolving process as better and new measures become available. The use of experiential coach knowledge could aid in the informed selection of constraints, however experiential knowledge is dependent on the individual, subject to biases and the environment in which it is applied. Future studies may examine the frequency of rule occurrence in a defined period of time (19). For instance, a SSG played in a small area may have a higher frequency of disposals per minute, compared to a larger area. Given the constraints sampled in the present study are discrete in nature, fuzzy approaches or similar methodologies could be used to link match events and spatiotemporal data, allowing the assessment of more constraints and insights into training representativeness.

Conclusion

Disposals are influenced by the interaction of constraints in training and competition environments in elite AF. Variation exists in the frequency whereby disposals occur under specific constraints across the competition matches, match simulations and SSGs. Although training and competition environments differed, greater levels of representativeness existed between match simulations and competition matches compared to SSGs and competition matches. These insights can aid the comprehension of how constraints interact to shape the emergence of specific disposals and their effectiveness, affording practitioners with a platform for the development and measurement of representative training tasks. The analytical techniques applied in the present study are not limited to AF and may assist in designing representative training tasks across other sports via the consideration of constraint interaction.

Importantly, this study provides a methodological advancement in the measurement of constraint influence, frequency and accounting for the continuous nature of sport.

Disclosure Statement: The authors report no conflict of interest

References

1. Pinder R, Davids K, Renshaw I, Araújo D. Representative learning design and functionality of research and practice in sport. Journal of Sport and Exercise Psychology. 2011;33(1):146-55.
2. Brunswik E. Perception and the representative design of psychological experiments: Univ of California Press; 1956.
3. Newell KM. Constraints on the development of coordination. Motor development in children: Aspects of coordination and control. 1986;34:341-60.
4. Davids K, Button C, Bennett S. Dynamics of skill acquisition: A constraints-led approach: Human Kinetics; 2008.
5. Woods CT, McKeown I, Shuttleworth RJ, Davids K, Robertson S. Training programme designs in professional team sport: An ecological dynamics exemplar. Human movement science. 2019;66:318-26.
6. Robertson S, Spencer B, Back N, Farrow D. A rule induction framework for the determination of representative learning design in skilled performance. Journal of Sport Science. 2019.
7. Araújo D, Davids K. The (Sport) Performer-Environment System as the Base Unit in Explanations of Expert Performance. Journal of Expertise/December. 2018;1(3).
8. McLean S, Hulme A, Mooney M, Read GJM, Bedford A, Salmon PM. A systems approach to performance analysis in women's netball: Using Work Domain Analysis to model elite netball performance. Frontiers in Psychology. 2019;10:201.
9. Vaughan J, Mallett CJ, Davids K, Potrac P, López-Felip MA. Developing Creativity to Enhance Human Potential in Sport: A Wicked Transdisciplinary Challenge. Frontiers in psychology. 2019;10:2090.
10. Browne PR, Sweeting AJ, Davids K, Robertson S. Prevalence of interactions and influence of performance constraints on kick outcomes across Australian Football tiers: Implications for representative practice designs. Human movement science. 2019:621-30.
11. Handford C, Davids K, Bennett S, Button C. Skill acquisition in sport: Some applications of an evolving practice ecology. Journal of sports sciences. 1997;15(6):621-40.
12. Araújo D, Davids K, Bennett SJ, Button C, Chapman G. Emergence of sport skills under constraints. In: Williams AM, Hodges NJ, editors. Skill acquisition in sport: Research, theory and practice: Routledge; 2004. p. 409.
13. Pocock C, Bezodis NE, Davids K, North JS. Hot hands, cold feet? Investigating effects of interacting constraints on place kicking performance at the 2015 Rugby Union World Cup. European journal of sport science. 2018:1-8.
14. Ometto L, Vasconcellos FV, Cunha FA, Teoldo I, Souza CRB, Dutra MB, et al. How manipulating task constraints in small-sided and conditioned games shapes emergence of individual and collective tactical behaviours in football: A systematic review. International Journal of Sports Science & Coaching. 2018;13(6):1200-14.
15. Canton A, Torrents C, Ric A, Gonçalves B, Sampaio JE, Hristovski R. Effects of temporary numerical imbalances on collective exploratory behaviour of young and professional football players. Frontiers in psychology. 2019;10:1968.
16. Hristovski R, Davids K, Araújo D, Passos P. Creativity in sport and dance: ecological dynamics on a hierarchically soft-assembled perception–action landscape. Complex systems in sport: Routledge; 2013. p. 287-300.

17. Gonçalves B, Esteves P, Folgado H, Ric A, Torrents C, Sampaio J. Effects of pitch area-restrictions on tactical behavior, physical, and physiological performances in soccer large-sided games. The Journal of Strength & Conditioning Research. 2017;31(9):2398-408.
18. Aguiar M, Botelho G, Lago C, Maças V, Sampaio J. A review on the effects of soccer small-sided games. Journal of human kinetics. 2012;33:103-13.
19. Ireland D, Dawson B, Peeling P, Lester L, Heasman J, Rogalski B. Do we train how we play? Investigating skill patterns in Australian football. Science and Medicine in Football. 2019:1-10.
20. Morgan S, editor Detecting Spatial Trends in Hockey Using Frequent Item Sets. Proceedings of the 8th International Symposium on Computer Science in Sport; 2011.
21. Hahsler M, Buchta C, Gruen B, Hornik K. Package 'arules'. 2018.
22. Browne PR, Morgan S, Bahnisch J, Robertson S. Discovering patterns of play in netball with network motifs and association rules. International Journal of Computer Science in Sport. 2019;18(1):64-79.
23. Wickham H, Francois R, Henry L, Müller K. dplyr: A Grammar of Data Manipulation. R package version 0.4. 3. R Found Stat Comput, Vienna https://CRAN R-project org/package= dplyr. 2015.
24. Wickham H, Henry L. tidyr: easily tidy data with "spread ()" and "gather ()" functions. R package version 0.8. 0. URL https://CRAN. R-project. org/package= tidyr [accessed 14 May 2018]; 2018.
25. Klusemann MJ, Pyne DB, Foster C, Drinkwater EJ. Optimising technical skills and physical loading in small-sided basketball games. Journal of sports sciences. 2012;30(14):1463-71.
26. Humberto Almeida C, Paulo Ferreira A, Volossovitch A. Manipulating task constraints in small-sided soccer games: Performance analysis and practical implications. The Open Sports Science Journal. 2012;5(1).
27. Farrow D, Robertson S. Development of a skill acquisition periodisation framework for high-performance sport. Sports Medicine. 2017;47(6):1043-54.
28. Corbett DM, Bartlett JD, O'connor F, Back N, Torres-Ronda L, Robertson S. Development of physical and skill training drill prescription systems for elite Australian Rules football. Science and Medicine in Football. 2018;2(1):51-7.

Figure Captions

Figure 1. Variation in levels of support (A) and confidence (B) of each Rule ID match simulations and Small Sided Games relative to competition matches. Where zero is equal to competition matches. Positive values reflect greater values for competition matches.

Figure 2. The observed frequency of effectiveness of the third disposal following two sequential disposals across competition matches A) competition match B) match simulation C) Small Sided Games. Values expressed as percentages (%).

Note: The scale moves from orange to blue with the deeper the hue the greater observed frequency of an effective third disposal. Blank sections are those which did not have two sequential passes. Grey circles reflect those sequences of passes which did not continue to a third disposal.

Figure 3. Density plot of the observed frequency of effectiveness of the third disposal following two sequential disposals across competition matches (green), match simulations (red) and Small Sided Games (blue).

Figure 1

Click here to download Figure Figure1.tif

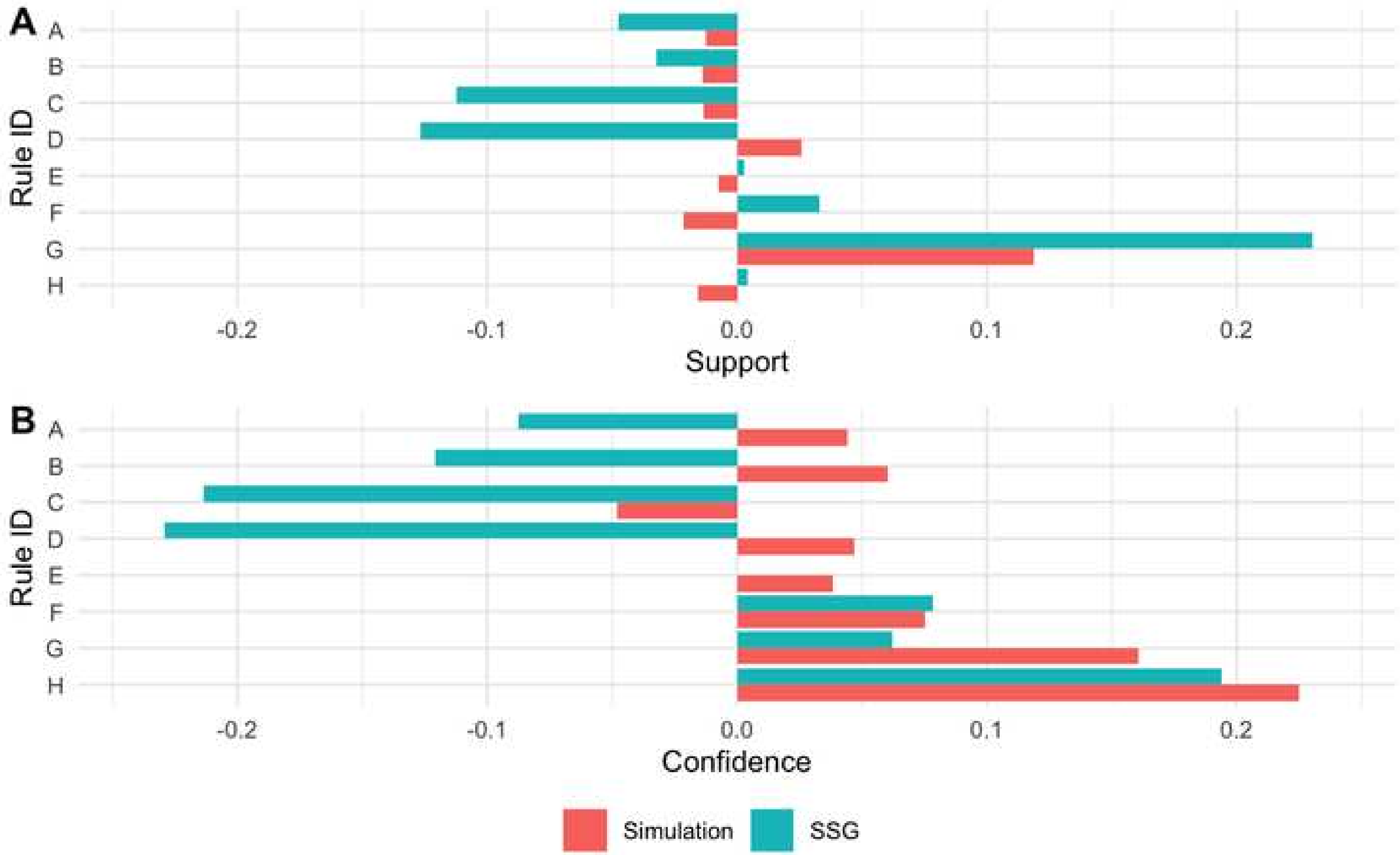

Figure 2 Click here to download Figure Figure2.tif

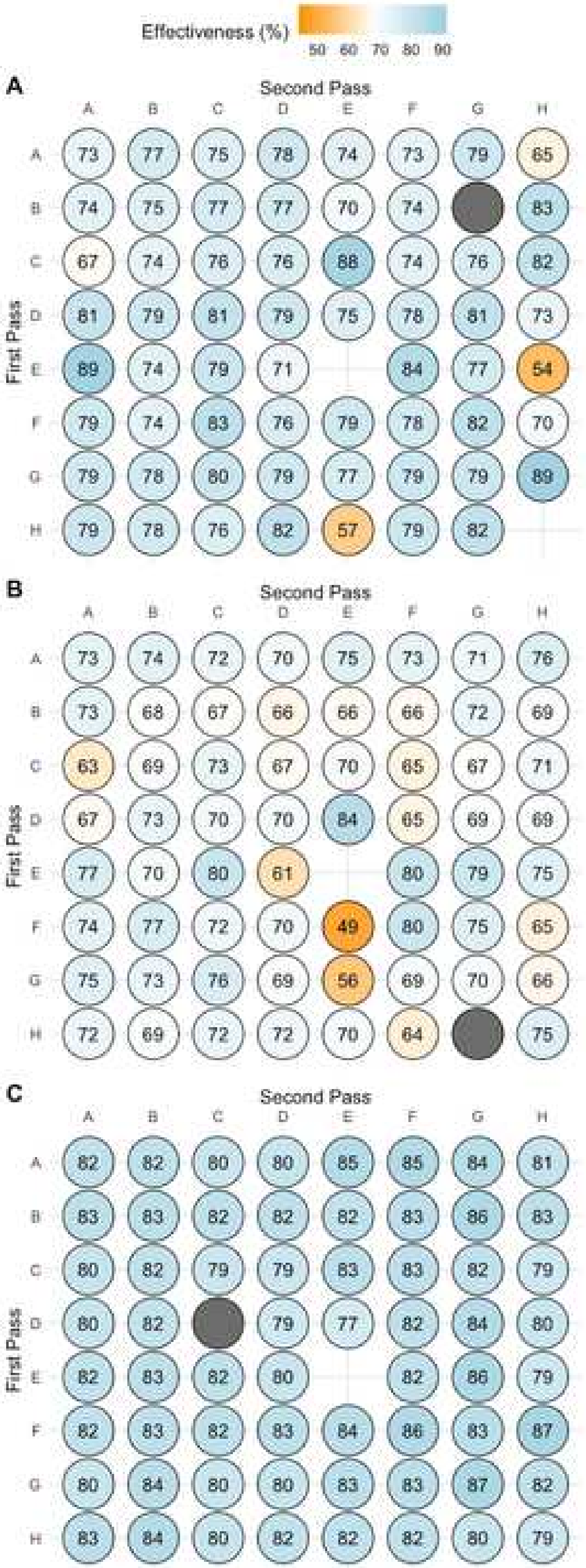

Figure 3

Click here to download Figure Figure3.tif

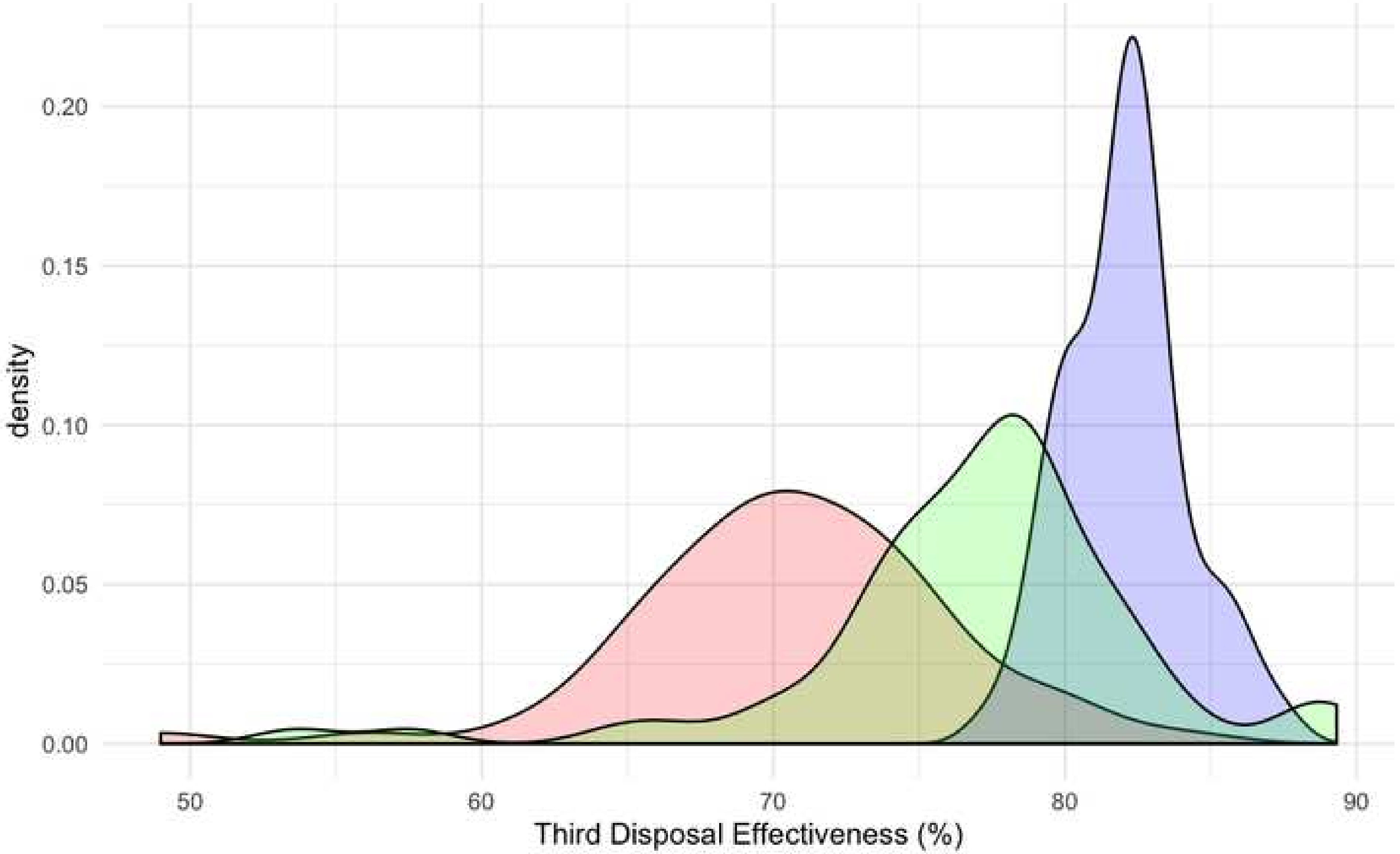

De-identified data

Click here to access/download
Supporting Information
Deidentified_Data.csv
